# Vascular endothelial microparticles-incorporated microRNAs are altered in patients with diabetes mellitus

**DOI:** 10.1186/s12933-016-0367-8

**Published:** 2016-03-22

**Authors:** Felix Jansen, Han Wang, David Przybilla, Bernardo S. Franklin, Andreas Dolf, Philipp Pfeifer, Theresa Schmitz, Anna Flender, Elmar Endl, Georg Nickenig, Nikos Werner

**Affiliations:** Medizinische Klinik und Poliklinik II, Universitätsklinikum Bonn, 53127 Bonn, Germany; Institute for Innate Immunity, Universitätsklinikum Bonn, 53127 Bonn, Germany; Institut für Molekulare Medizin, Universitätsklinikum Bonn, 53127 Bonn, Germany

**Keywords:** microRNA, Microparticles, Diabetes mellitus, Vascular

## Abstract

**Background:**

Circulating microRNAs (miRs) are differentially regulated and selectively packaged into microparticles (MPs). We evaluated whether diabetes mellitus alters circulating vascular and endothelial MP-incorporated miRs expression levels.

**Methods and results:**

Circulating MPs were isolated from 135 patients with or without diabetes mellitus type II and characterized using flow cytometer and electron microscope. Nine miRs involved in the regulation of vascular performance—miR-126, miR-222, miR-let7d, miR-21, miR-30, miR-92a, miR-139, miR-199a and miR-26a—were quantified in circulating MPs by reverse transcription polymerase chain reaction. Among those, miR-126 and miR-26a were significantly reduced in diabetic patients compared to non-diabetic patients. Patients with low miR-26a and miR-126 levels were at higher risk for a concomitant coronary artery disease. MP-sorting experiments showed that endothelial cells were the major cell sources of MPs containing miR-126 and miR-26a, respectively. Finally, in accordance with our clinical results, in vitro experiments revealed that hyperglycemia reduces the packaging of miR-126 and miR-26a into EMPs.

**Conclusion:**

Diabetes mellitus significantly alters the expression of vascular endothelial miRs in circulating endothelial MPs with potential implications on vascular heath.

**Electronic supplementary material:**

The online version of this article (doi:10.1186/s12933-016-0367-8) contains supplementary material, which is available to authorized users.

## Background

MicroRNAs (miRs) are small (22-nucleotide) noncoding RNAs regulating gene expression at the posttranscriptional level by binding to the target mRNA, leading either to mRNA degradation or to translational repression [1]. miRs have emerged as key regulators of several physiological and pathophysiological processes in cardiovascular (CV) and metabolic health and disease [2, 3]. Besides their intracellular function, recent studies have demonstrated that miRs can be exported or released by cells and circulate in the blood in a remarkably stable form [4]. The discovery of circulating miRs opens up fascinating possibilities to use circulating miR patterns as biomarkers for CV and metabolic diseases [5, 6]. Altered levels of circulating miRs have been reported in patients with heart failure, coronary artery disease (CAD), and diabetes [7–9].

Recent findings have emerged that microparticles (MPs) represent major protective transport vehicles for miRs by separating them from circulating ribonuclease (RNase) [10]. Moreover, increasing evidence suggests that MP-associated miRs not only represent passively released cellular debris, but may also contribute to intercellular signaling mechanisms [11]. In this context, we and others have demonstrated that endothelial cell-derived MPs mediate vascular protection and endothelial regeneration in a miR-126-dependent mechanism [12, 13]. Importantly, previous data revealed that the biological content and functional effect of MPs depends on the condition of the releasing cell. Hyperglycemic conditions in vitro significantly changed miRs expression levels in MPs and subsequently altered their biological effect on target cells [12, 14]. Taken together, these findings suggest that circulating MP-packaged miRs, in addition to their function as biomarkers, represent functional mediators in vascular and metabolic diseases.

Diabetes mellitus is characterized by increased blood glucose levels and represents a major risk factor for cardiovascular morbidity and mortality. As a consequence of endothelial activation and dysfunction, diabetic patients show increased plasma level of circulating MPs [15]. Moreover, elevated levels of circulating EMPs are associated with vascular dysfunction in diabetic patients and MPs derived from hyperglycemic cells fostered atherogenesis, suggesting an active role of MPs in diabetic complications [16, 17].

Several studies showed promising results for the use of circulating miRs as potential biomarker in diabetic patients. However, whether diabetes mellitus is associated with changes in miRs expression pattern in circulating MPs is unknown. As miR-containing MPs regulate vascular function and disease progression, a detailed exploration of miRs expression in circulating MPs in patients with and without diabetes would be of high interest to understand the pathogenesis of vascular complications in diabetic patients and develop novel therapies opportunities.

In a translational approach, we first describe that diabetes mellitus alters vascular miRs expression levels in circulating MPs. These findings provide novel evidence for a potential role of miR-containing MPs in the regulation of vascular integrity in patients with diabetes mellitus.

## Methods

### Study subjects

Between August 2012 and July 2013, 141 patients presenting in our outpatient department were screened for inclusion in the study. Six patients with clinical presentation of acute or subacute myocardial infarction were excluded from the study. Patients with malignant, inflammatory diseases, or severe hepatic or renal dysfunction were also excluded from the study. Informed consent was obtained from all patients and the ethics committee of the University of Bonn approved the study protocol. Based on previous medical reports, patients were either grouped in the diabetes mellitus group (DM, n = 55) or the non-diabetic group (NDM group, n = 80). All patients included in DM group took either oral antidiabetic drugs or used subcutaneous insulin application.

### Preparation of blood samples

Venous blood was drawn under sterile conditions from the cubital vein and was buffered using sodium citrate (for MPs quantification) or ethylenediaminetetraacetic acid (EDTA, for miR analysis). Additional blood samples for routine analyses were obtained. Blood was centrifuged at 1500*g* for 15 min followed by centrifugation at 13,000*g* for 2 min to generate platelet-deficient plasma. The deprived plasma samples were immediately stored in −80 °C. Annexin V/CD 31 positive microparticles level were measured freshly with flow cytometry by using Annexin V-FITC and CD31-PE (BD Pharmingen). Platelet-deficient plasma was stored in −80 °C until miR level were analyzed.

### Microparticles collection and RNA isolation

RNA was isolated from circulating MPs by using TRIzol-based miR isolation protocol. 250 μl total plasma was centrifuged at 20,000*g* for 30 min at 4 °C to pellet circulating MPs as previously described [18]. The pellet was diluted in 250 μl RNase-free water and then diluted in 750 μl TRIzol^®^ LS in order to measure MPs miRs levels. *Caenorhabditis elegans* miR-39 (cel-miR-39, 5 nM, Qiagen) was spiked in TRIzol for normalization of miR content as described [19]. To increase the yield of small RNAs, the RNA was precipitated in ethanol at −20 °C overnight with glycogen (Invitrogen).

### Sorting of microparticles subspecies

For sorting of MPs subspecies, 250 µl platelet-free plasma was stained with CD31-PE, and CD42b-APC (BD Pharmingen) and the corresponding isotype and negative controls. Stained plasma was incubated for 45 min in dark at room temperature according to the manufactures suggestions.

To sort MPs subspecies, a FACSAria™ III Flow Cytometer (BD Biosciences) was used. Vesicles between 100–1000 nm in diameter were gated for sorting. CD31+/CD42b−, CD31+/CD42b+ and CD31−/CD42b− MPs were gated, sorted and collected as shown in Additional file 1. RNAse-free water was added to the sorted MPs to reach a total volume of 250 μl, which was diluted in 750 μl TRIzol^®^ LS in order to measure MPs miRs levels. *C. elegans* miR-39 (cel-miR-39, 5 nM, Qiagen) was spiked in TRIzol for normalization of miRs content as described [20]. To increase the yield of small RNAs, the RNA was precipitated in ethanol at −20 °C overnight with glycogen (Invitrogen).

### Quantification of miRs by quantitative PCR

RNA was quantified using Nanodrop spectrophotometer (Nanodrop Technologies Inc). 10 ng of the total RNA was reversely transcribed using TaqMan^®^ microRNA reverse transcription kit (Applied Biosystems) according to the manufactures protocol. miR-126, miR-222, miR-let7d, miR-21, miR-30, miR-92a, miR-139, miR-199a and miR-26a in circulating MPs were detected by using TaqMan^®^ microRNA assays (Applied Biosystems) on a 7500 HT Real-Time PCR machine (Applied Biosystems). Cel-miR-39 was used as an endogenous control. For all miRs, a Ct value above 40 was defined as undetectable. Delta Ct method was used to quantify relative microRNA expression. Values were normalized to cel-miR-39 and are expressed as 2^−ddct^ log 10. For all PCR experiments, samples were run in triplicates.

### Cell culture and endothelial microparticles generation

Human coronary artery endothelial cells (HCAEC, PromoCell) were cultured in endothelial cell growth media with endothelial growth media Supplement Mix (Promocell) under standard cell culture conditions (37 °C, 5 % CO_2_). Cells of passage 4–7 were used when 70–80 % confluent. Endothelial microparticles (EMPs) were generated from HCAEC as previously described with minor changes [21]. Briefly, confluent cells were starved by subjecting to basal media without growth media supplements for 24 h to induce apoptosis. After starvation, supernatant of apoptotic HCAEC was collected and centrifuged at 1500*g* for 15 min to remove cell debris. The supernatant was centrifuged (20,000*g*, 40 min) to pellet EMPs. The obtained EMPs were washed in sterile phosphate buffered saline (PBS, pH 7.4) and pelleted again at 20,000*g* for 40 min. In order to generate EMPs from endothelial cells under hyperglycemic conditions, confluent HCAEC were stimulated with 30 mM glucose for 72 h [22] and then subjected to basal media without growth media supplements for 24 h to generate EMPs. Microparticles derived from glucose-treated endothelial cells were defined as “high glucose” EMPs (hgEMPs). Pelleted EMPs were resuspended in sterile PBS and used freshly.

### Electron microscope

MPs were isolated using 20,000*g* ultracentrifugation as described previously [20]. The obtained pellet was fixed in 3 % glutaraldehyde PBS overnight at 4 °C. The pellet was then washed with 0.1 M cacodylate buffer, postfixed in 2 % OsO4, washed again with 0.1 M cacodylate buffer and dehydrated in graded ethanol. The sample was embedded in Epon-pur and 50 nm sections were prepared on copper grids. Samples were visualized on a Philips CM 10 electronic microscope and analyzed with analySiS software (Olympus).

### Flow cytometry analysis on EMPs

In vitro generated and pelleted EMPs were suspended in 100 µl annexin V-binding buffer (10 mM HEPES, pH 7.4, 140 mM NaCl, 2.5 mM CaCl_2_) with and without calcium as control. 5 µl annexin V-FITC (BD Biosciences) was added. After incubation for 15 min at room temperature, diluted EMPs were centrifuged for 20 min at 20,000*g*, washed with sterile PBS and re-centrifuged. The pelleted EMPs were resuspended in 100 µl annexin V-binding buffer and 4 µl CD31-PE (BD Biosciences) or isotype control was added. After incubation for 30 min at room temperature, diluted EMPs were centrifuged and washed as described above. Pelleted EMPs were resuspended in 200 µl annexin V-binding buffer and analyzed with FACS BD LSR II. To evaluate the size of EMPs, the following fluorescent reference beads were used: nile red particles 0.7–0.9 µm (Spherotech), nile red particles 2 µm (Spherotech), BD Calibrate 3 Beads 6 µm (BD Biosciences).

### MicroRNA expression in vitro

Total RNA was isolated out of EMPs, hgEMPs, HCAEC and hgHCAEC by TRIzol (Invitrogen) extraction method according to instruction of the manufacturer. To increase the yield of small RNAs, the RNA is precipitated in ethanol at −20 °C overnight with glycogen (Invitrogen). RNA is quantified using Nanodrop spectrophotometer. Then, 10 ng of the total RNA was reversely transcribed using TaqMan^®^ microRNA reverse transcription kit (Applied Biosystems) according to the manufacturers protocol. Taqman microRNA assays (Applied Biosystems) were used to measure miR-126 and miR-26a levels on a 7500 HT Real-Time PCR machine (Applied Biosystems). RNU-6b was used as an endogenous control. Delta Ct method was used to quantify relative microRNA expression.

### Statistical analysis

Continuous variables were tested for normal distribution with Kolmogorov–Smirnov test. Normally distributed continuous variables were presented as mean ± SD. Mann–Whitney U test was used to analyze variables with skewed distribution. Means between two categories were compared with the two-tailed, unpaired Student’s *t* test. The Chi square test was used for categorical data that result from classifying objects. Binary logistic regression was applied to identify factors that were independently associated with miR-126 and miR-26a. Statistical significance was assumed when the null hypothesis could be rejected at p < 0.05. Statistical analysis was performed with IBM SPSS Statistics version 20 (USA).

## Results

### Baseline characteristics

A total of 135 patients with (DM, n = 55) or without diabetes mellitus (NDM, n = 80) were enrolled in the study. There was no difference regarding age and sex between the groups. DM patients had more frequently a concomitant arterial hypertension (p = 0.024), a higher body mass index (p = 0.001) and coronary artery disease (p = 0.009). Regarding the medication, DM patients took more frequently calcium channel blockers (p = 0.019). As expected, DM patients had higher fasting blood glucose levels (p = 0.0001) and higher HbA1c values (p = 0.0001). DM patients showed reduced HDL (p = 0.009) and LDL (p = 0.03) levels. Furthermore, DM was associated with a significantly higher number of circulating annexin V-positive MPs (p = 0.014, Table 1).

**Table 1:** Baseline characteristics of the study population

Characteristic	Total (n = 135)	DM (n = 55)	NDM (n = 80)	p value
Age in years	66.4 ± 10.9	67.6 ± 10.6	65.5 ± 11.1	0.279
Gender no. (%)				0.711
Female	45 (33.3 %)	17 (30.9 %)	28 (35 %)	
Male	90 (66.7 %)	38 (69.1 %)	52 (65 %)	
Cardiovascular risk factors no. (%)				
Arterial hypertension	110 (81.5 %)	50 (90.9 %)	60 (75 %)	*0.024*
Hyperlipoproteinemia	71 (52.6 %)	29 (52.7 %)	42 (52.5 %)	0.979
Family history of CAD	40 (29.6 %)	14 (25.5 %)	16 (20 %)	0.529
Smoking	29 (21.5 %)	10 (18.2 %)	19 (23.8 %)	0.525
Body mass index kg/m^2^	28.4 ± 5.2	30.5 ± 5.4	26.9 ± 4.5	*0.001*
Medical history no. (%)				
Previous MI (6 months)	40 (29.6 %)	16 (29.1 %)	24 (30 %)	0.910
Previous bypass	15 (11.1 %)	8 (14.5 %)	7 (8.8 %)	0.404
Previous stroke	7 (5.2 %)	3 (5.5 %)	4 (5 %)	0.907
Chronic kidney disease	4 (3.0 %)	2 (3.6 %)	2 (2.5 %)	0.702
Coronary artery disease no. (%)				*0.009*
3 vessels	49 (36.3 %)	24 (44.4 %)	25 (31.7 %)	
2 vessels	37 (27.4 %)	20 (37.0 %)	17 (21.5 %)	
1 vessel	15 (11.1 %)	3 (5.6 %)	12 (15.2 %)	
Left ventricular ejection fraction (%)	57.2 ± 12.2	60.0 ± 12.8	57.4 ± 11.8	0.831
Medication on admission no. (%)				
ACE inhibitors	85 (63.0 %)	35 (63.6 %)	50 (62.5 %)	0.893
Angiotensin receptor blockers	29 (21.5 %)	13 (23.6 %)	16 (20 %)	0.672
Beta blockers	115 (85.2 %)	45 (81.8 %)	70 (87.5 %)	0.461
Calcium channel blockers	28 (20.7 %)	17 (30.9 %)	11 (13.8 %)	*0.019*
Diuretics	62 (45.9 %)	27 (49.1 %)	35 (43.8 %)	0.600
Statins	109 (80.7 %)	45 (81.8 %)	64 (80 %)	0.828
Nitrates	5 (3.7 %)	1 (1.8 %)	4 (5 %)	0.648
Aspirin	115 (85.2 %)	47 (85.5 %)	58 (72.5 %)	0.093
Laboratory parameters				
Glucose mg/dL	126.4 ± 58.6	167.1 ± 72.3	99.0 ± 19.2	*0.0001*
HbA1c (%)	6.4 ± 1.0	7.2 ± 1.2	5.9 ± 0.4	*0.0001*
Serum creatinine mg/dL	0.98 ± 0.33	1.01 ± 0.33	0.97 ± 0.32	0.645
Glomerular filtration rate mL/min	65.1 ± 11.0	64.3 ± 12.4	65.7 ± 9.9	0.463
Triglycerides mg/dL	166.2 ± 204.5	199.4 ± 302.8	144.4 ± 84.2	0.137
Cholesterol mg/dL	184.645 ± 48.8	173.2 ± 44.6	192.5 ± 50.2	*0.023*
HDL cholesterol mg/dL	49.9 ± 17.7	45.2 ± 16.6	53.2 ± 17.8	*0.009*
LDL cholesterol mg/dL	110.3 ± 37.3	102.0 ± 34.2	116.0 ± 38.4	*0.030*
C-reactive protein mg/L	4.4 ± 6.5	4.5 ± 5.8	4.3 ± 7.0	0.826
Leucocytes 10^9^/L	7.3 ± 2.1	7.3 ± 1.7	7.2 ± 2.3	0.807
Concentration of Annexin V + MP/µl	778.72 ± 1439.4	1144.0 ± 1849.4	526.2 ± 1009.1	*0.014*

Continuous data are expressed as mean ± standard deviation and categorical data are expressed as frequencies. Chronic kidney disease was defined as a glomerular filtration rate <60 mL/min

*DM* diabetes mellitus, *NDM* non diabetes mellitus, *CAD* coronary artery disease, *MI* myocardial infarction, *ACE* angiotensin-converting enzyme, *LDL* low-density lipoprotein, *HDL* high-density lipoprotein, *MP* microparticle

Italic values indicate significance of p value (p < 0.05)

miR selection and detection in circulating MPs.

Nine vascular and endothelial cell–expressed miRs that have been shown to be involved in the pathogenesis of diabetes mellitus were chosen to compare their expression levels in DM and NDM patients: miR-126, miR-222, miR-let7d, miR-21, miR-30, miR-92a, miR-139, miR-199a and miR-26a. Because previous studies strongly suggest that circulating miRs are selectively packaged into MPs, levels of analyzed miRs were measured in circulating MPs in all patients.

### Circulating microparticles characterization

**Fig. 1:**
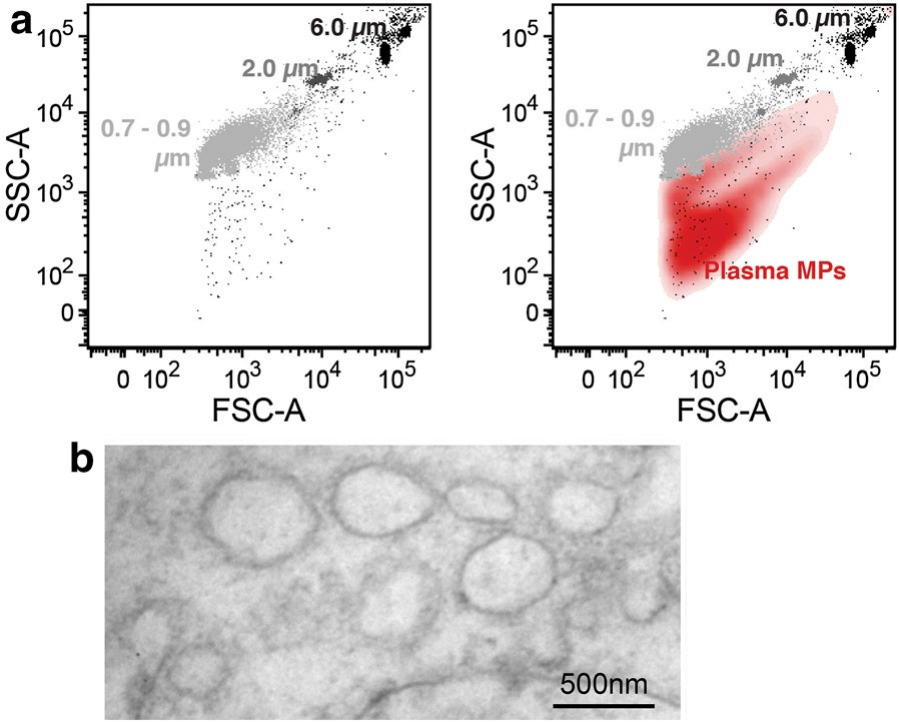
Circulating MPs characterization. **a** Circulating MPs were collected by 20,000*g* ultracentrifugation of platelet-deficient plasma. Fluorescent polystyrene particles (0.7–0.9, 2 and 6 µm) were used to evaluate the size of isolated MPs. In flow cytometer, analyzed MPs (*red*) had a size <1 μm as demonstrated using fluorescent polystyrene particles. **b** The obtained pellet after 20,000*g* ultracentrifugation was fixed in 3 % glutaraldehyde PBS overnight at 4 °C and embedded in Epon-pur. 50 nm sections were prepared on copper grids. Samples were visualized on a Philips CM 10 electronic microscope and analyzed with analySiS software (Olympus)

Isolated MPs were characterized regarding their size using electron microscopy and flow cytometer. Characterization experiments revealed that the vast majority of isolated MPs had a size between 0.1 and 1 µm in diameter (Fig. 1a, b).

miR expression in circulating MPs in DM and NDM patients.

**Fig. 2:**
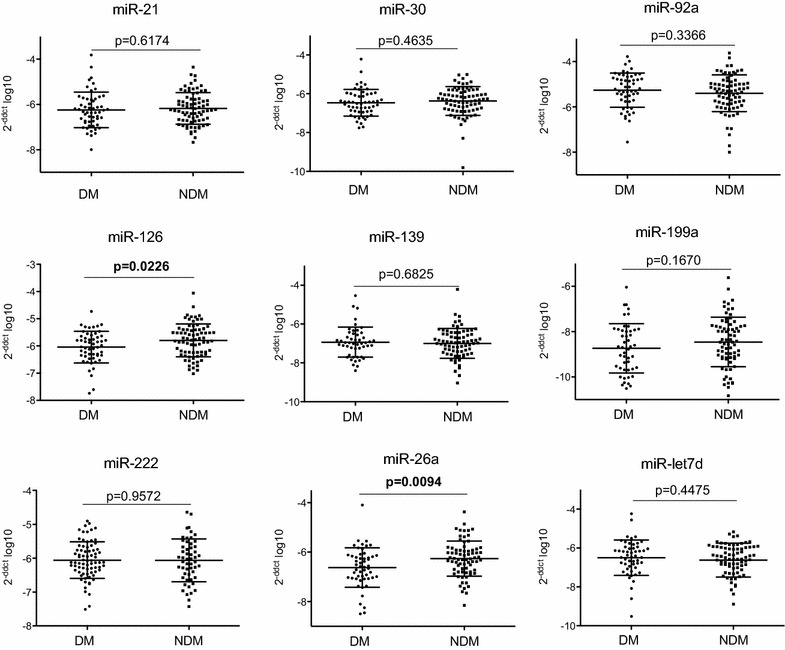
miRs expression in circulating MPs in DM and NDM patients. Delta Ct method was used to quantify relative microRNA expression. Values were normalized to cel-miR-39 and are expressed as 2^−[CT(microRNA)−CT(cel–miR−39)]^ log10

Analysis of miR expression pattern in isolated MPs from DM and NDM patients showed no difference in miR-222, miR-let7d, miR-21, miR-30, miR-92a, miR-139 and miR-199a. In contrast, miR-26a and miR-126 were significantly reduced in DM patients compared to NDM patients (Fig. 2).

Comorbidities such as hypertension and CAD or medication affect circulating miR levels. Binary logistic regression analysis demonstrated that changes in expression levels of miR-126 and miR-26a were associated with a concomitant CAD, but independent of other comorbidities. Regarding medication intake, there was a significant association of miR-126 levels and statin intake, whereas miR-126 and miR-26a levels were independent of all other drugs (Tables 2, 3). To explore the association of miR-26a and miR-126 expression with the occurrence of a concomitant CAD, we categorized the study population into two groups according to the median of miR-26a and miR-126 expression. Importantly, patients with reduced miR-26a and miR-126 expression levels were at higher risk for the occurrence of a concomitant CAD (Table 4).

**Table 2:** Association of miR-26a levels with baseline characteristics

	Exp(B) (95 % CI)	p value
Age	0.981 (0.932–1.033)	0.465
Male sex	1.306 (0.409–4.174)	0.652
Body mass index	1.062 (0.950–1.186)	0.291
Coronary artery disease	2.320 (1.047–5.138)	*0.038*
Arterial hypertension	0.632 (0.205–1.953)	0.426
Hyperlipoproteinemia	1.179 (0.445–3.123)	0.740
Smoking	0.808 (0.247–2.641)	0.725
Chronic kidney disease	1.101 (0.081–14.961)	0.943
Angiotensin Converting Enzyme Inhibitors (ACEI)	1.404 (0.440–4.482)	0.566
Angiotensin receptor blockers (ARB)	0.970 (0.269–3.495)	0.963
Calcium channel blockers (CCB)	1.566 (0.565–4.339)	0.389
Diuretics	0.551 (0.252–1.205)	0.135
Statins	0.615 (0.185–2.037)	0.426
Nitrates	1.492 (0.215–10.347)	0.685
Aspirin	0.664 (0.196–2.248)	0.510
Clopidogrel	0.675 (0.293–1.558)	0.358

The coefficient of the continuous variables was relative to 1-U differences. Binary logistic regression according to the median of miR-26a level

*Exp(B)* exponentiation of the B coefficient, *miR* microRNA

Italic value indicates significance of p value (p < 0.05)

**Table 3:** Association of miR-126 levels with baseline characteristics

	Exp(B) (95 % CI)	p value
Age	0.981(0.937–1.026)	0.402
Male sex	1.480 (0.534–4.101)	0.451
Body mass index	1.031 (0.963–1.102)	0.381
Coronary artery disease	2.444 (1.086–5.051)	*0.031*
Arterial hypertension	0.410 (0.133–1.266)	0.121
Hyperlipoproteinemia	0.831 (0.354–1.953)	0.671
Smoking	1.227 (0.411–3.667)	0.714
Chronic kidney disease	1.393 (0.106–18.389)	0.801
Angiotensin converting enzyme inhibitors (ACEI)	0.803 (0.241–2.682)	0.722
Angiotensin receptor blockers (ARB)	0.634 (0.166–2.423)	0.505
Calcium channel blockers (CCB)	1.237 (0.462–3.310)	0.673
Diuretics	0.501 (0.227–1.103)	0.086
Statins	0.158 (0.040–0.632)	*0.009*
Nitrates	3.256 (0.263–40.306)	0.358
Aspirin	2.075 (0.550–7.825)	0.281
Clopidogrel	0.681 (0.295–1.573)	0.368

The coefficient of the continuous variables was relative to 1-U differences. Binary logistic regression according to the median of miR-126 level

*Exp(B)* exponentiation of the B coefficient, *miR* microRNA

Italic values indicate significance of p value (p < 0.05)

**Table 4:** Lower miR-26a and miR-126 levels are associated with the occurrence of coronary artery disease

	low level (n = 62)	high level (n = 63)	p value
miR-26a			
No CAD (n = 37)	13	24	0.049
CAD (n = 88)	49	39	

	low level (n = 63)	high level (n = 63)	p value
miR-126			
No CAD (n = 35)	12	23	0.046
CAD (n = 91)	51	40	

*Low level* below the median, *high level* above the median, *CAD* coronary artery disease, *miR* microRNA

### Endothelial MPs are the major source for circulating MP-bound miR-126 and miR-26

**Fig. 3:**
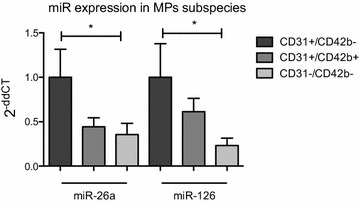
Analysis of microRNA in microparticle subspecies. Endothelial cell–derived (CD31+/CD42b−), platelet-derived (CD31+/CD42b+), and other cell–derived MPs (CD31−/CD42b−) were from 10 plasma samples of DM patients, and miR-126 and miR-26a expression were analyzed in MPs subspecies. **p < 0.01, n = 10. Relative quantification of miR expression was determined using the comparative CT method [2−ddCT, internal control: Cel-miR−39]. MPs indicate microparticles

Because circulating MPs compose different subspecies of membrane particles released from endothelium and blood cells, we sorted endothelial-, platelet-, and other cell-derived MPs using flow cytometer to explore the cellular origins of MP-bound miR-126 and miR-26a in DM patients. Overall, miR-126 and miR-26a showed the highest expression in CD31+/CD42b− endothelial cell-derived MPs, compared to CD31+/CD42b+ platelet-derived MPs and annexin V+/CD31−/CD42b− MPs (Fig. 3).

To explore whether CD31+/CD42b− MPs are the major source for other endothelial miRs as well, two other endothelial miRs—namely miR-199a and miR-let7d—were additionally analyzed in different MPs subsets. In contrast to miR-126 and miR-26a, RT-PCR experiments revealed no significant differences in miR-199a and miR-let7d expression in different MPs subsets, suggesting that miR-126 and miR-26a are selectively packaged into endothelial cell-derived MPs (Additional file 1).

### Hyperglycemia in vitro reduces endothelial MP-incorporated miR-126 and miR-26a expression

**Fig. 4:**
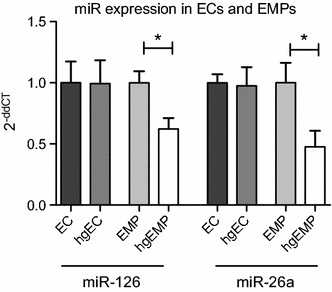
Hyperglycemia reduces miR-126 and miR-26a level in hgEMPs. miR-126 and miR-26a were was analyzed in ECs, hgECs, EMPs and hgEMPs. RNU6b served as endogenous control. *p < 0.05, n = 5–6

As MP-bound miR-126 and miR-26a were significantly reduced in DM patients and endothelial cell-derived MPs were shown to be the major source of miR-126 and miR-26-containing MPs, we finally explored the effect of hyperglycemic conditions in vitro on miRs expression on endothelial cells and endothelial MPs. In accordance with the clinical data, hyperglycemia significantly reduced the expression of miR-126 and miR-26a in endothelial cell-derived MPs without affecting the cellular level (Fig. 4).

Analysis of other endothelial miRs (miR-21, miR-30, miR-92a, miR-139, miR-222) revealed that hyperglycemia additionally, besides miR-26a and miR-126, reduced expression of miR-222, whereas expression of other miRs were not affected (Additional file 1).

Taken together, we provide evidence that diabetes mellitus affects circulating MP-incorporated vascular endothelial miRs expression level with potential implications for vascular health.

## Discussion

miRs are powerful regulators of cellular processes. Moreover, an increasing number of studies demonstrate that miRs can be detected in circulating blood and that these circulating miRs might be useful biomarkers in patients with metabolic diseases such as diabetes mellitus. Circulating MPs represent major transport vehicles for miRs by separating them from circulating RNase. Moreover, DM patients show elevated levels of circulating MPs, which might be actively involved in the progression of vascular dysfunction in diabetic conditions [16, 17]. However, so far the biological content of circulating MPs in patients with or without DM is vastly unknown.

### Role of miR-126 and miR-26a in vascular biology

In this study, we found that DM is associated with reduced endothelial miR-126 and miR-26a expression in circulating MPs. Previous clinical studies pointed out a potential implication of miR-126 in the context of cardiovascular and metabolic diseases. Analysis of circulating miRs in patients with CAD showed significantly reduced levels of miR-126 in patients with CAD in comparison with healthy controls [19]. Furthermore, plasma miRs profiling revealed a significant loss of miR-126 in patients with diabetes mellitus [9]. In circulating angiogenic early outgrowth cells and CD34+ peripheral blood mononuclear cells, intracellular miR-126 expression defined their regenerative capacity and was reduced in diabetic patients [23, 24]. Own data showed that patients with stable coronary artery disease and diabetes mellitus express reduced levels of circulating MP-bound miR-126 compared to non-diabetic patients [12]. Additional experiments revealed that miR-126-containing endothelial cell-derived MPs promoted vascular regeneration, which was abrogated in MPs collected under hyperglycaemic conditions [12]. These findings strongly indicate a crucial role for miR-transporting MPs in the regulation of vascular health, which is altered under diabetic conditions. Furthermore, we found that MP-bound miR-222 was reduced in endothelial cell-derived MPs under hyperglycaemic conditions [14]. However, in the present study, there was no difference in circulating miR-222 levels between DM and NDM patients. These findings suggest that freely circulating miRs levels and MP-bound miRs can be regulated independently and differently in diabetic conditions.

miR-26a is expressed, besides others, in endothelial cells and has been shown to prevent endothelial cell apoptosis by directly targeting TRPC6 in atherosclerotic mice [25]. In the setting of diabetes, it has been recently shown that liver-specific miR-26a plays a crucial role in the regulation of insulin sensitivity and glucose metabolism in obese mice and human. Of note, similar to our findings, miR-26a expression was significantly reduced in obese human and mice compared to healthy controls [26]. Given that miR-26a inhibits endothelial apoptosis, and miR-containing MPs can affect target cell biology, one may speculate that MPs with low miR-26a expression levels as occurring in diabetic patients might have a reduced protective effect on target cells. Impaired angiogenesis is one main microangiopathic complication in patients with diabetes mellitus. In this context, miR-26a has been shown to regulate pathological and physiological angiogenesis by targeting BMP/SMAD1-signaling. Inhibition of miR-26a induced robust angiogenesis within 2 days, an effect associated with reduced myocardial infarct size and improved heart function [27].

Our data show that MP-incorporated miR-26a expression is reduced in patients with DM. Based on these findings and the before mentioned publication, one would suggest that lower levels of miR-26a in circulating MPs in DM patients would rather promote angiogenesis in target cells. If this comes true, this could be a compensatory mechanism from endothelial cells to release MPs with a proangiogenic message as an attempt to decelerate DM-associated impairment of angiogenesis.

MP-sorting experiments showed that endothelial cells were found to be the major cell sources of MPs containing miR-126 and miR-26a in diabetic patients. In accordance with these data, circulating MP-bound miR-126 was mainly expressed in circulating endothelial cell-derived MPs in patients with stable coronary artery disease, whereas miR-199a was primarily detectable in platelet-derived MPs [20]. However, another study found platelets as major contributor to circulating miR-126 signatures in patients with acute myocardial infarction [28]. These differences might be due to diverse patients collectives, different pathological conditions and/or variances in the used MPs isolation and miRs analysis protocols.

### Extracellular vesicles-incorporated miRs in intercellular communication

Circulating miRs in plasma can be transported within extracellular vesicles (exosomes, MPs, apoptotic bodies) [29] or bound to proteins (high-density lipoprotein, Ago-2) [30, 31]. Both routes provide remarkable stability and resistance to degradation from endogenous RNase activity. Previously, we found that endothelial cell-derived miR-126 and miR-199a were mainly expressed in circulating MPs, whereas miR-222, miR-21, miR-27, and miR-92a were detectable mainly in vesicle-free plasma. As MP-bound miRs, compared to freely circulating miRs, were shown to predict cardiovascular events in patients with stable coronary artery disease [32], we focused on analyzing the expression of MP-bound miRs in this study. A selective packaging of miRs into different plasma sub compartments was recently shown by Wang et al., who compared profiles of miRs in cell-derived vesicles (i.e., exosomes and MPs) with vesicle-free miRs (i.e., supernatant fraction after ultracentrifugation) and found that miRs profiles within and outside these vesicles were strikingly different [33].

The notion that miRs selectively packaged into MPs may play a crucial role in intercellular signaling is supported by an increasing experimental data [11, 34]. In this context, injected miRs containing apoptotic bodies were shown to be transported into atherosclerotic lesions, where they controlled the downstream target CXCL12 and promoted vascular protection. Furthermore, Hergenreider et al. described an atheroprotective communication mechanism between endothelial cells and vascular smooth muscle cells via endothelial cell-derived exosomes in a miR-143/145-dependent way. Taken together, these well-performed and convincing studies demonstrated the cardioprotective potential of intercellular communication mechanisms by miR-containing extracellular vesicles [13, 34].

Our study broaden these findings by demonstrating that not only cardiovascular, but also metabolic disorders like diabetes mellitus alter the expression of vascular miRs in circulating MPs.

Of note, miRs expression in diabetic vasculopathy is regulated by diverse factors. In this context, vitamin D has been described as important issue manipulating miRs expression in diabetic vasculopathy [35]. Furthermore, miR-1 and miR-208a were regulated dependent on the gender of examined mice in a model of streptozotocin-induced diabetes [36]. Diabetes and hyperlipidemia-induced inflammatory responses can upregulate the expression of connexins and Rho kinase by selective downregulation of the expression of miR-10a, miR-139b, miR-206, and miR-222 [37]. Exploration of circulating miRs as a biomarker revealed, in the setting of acute heart failure, that low circulating levels of miR-423-5p at presentation were associated with a poor long-term outcome [38]. Importantly, miRs can target several genes. That can be associated with undesired ‘off-target’ side effects, which has to be taken into consideration in general in miRs research [39].

## Limitations

This study has limitations. Only a selected number of miRs, based on previously published data, were analyzed. Moreover, although there is profound knowledge concerning the function of endothelial miR-126, the role of vascular miR-26a in diabetic conditions is largely unknown. Further exploration of MPs derived under normal and hyperglycaemic conditions containing different miR-26a levels is of importance to understand their role on vascular biology. Regarding the patients characterization, we did not prospectively collect the data for duration of all DM patients and can not give exact data on disease duration for DM patients.

In addition, exploration of selection and packaging mechanisms of miRs into MPs would be of interest to better comprehend the physiological and pathophysiological functions of miR-containing MPs in metabolic and vascular biology. Furthermore, the analysis of circulating miRs bound to other cargos than miRs (e.g. HDL or Ago proteins) would be of interest for future studies. Finally, the relatively small sample size limits the final conclusion that can be drawn from this study.

## Conclusions

Taken together, we show that diabetes mellitus significantly alters the expression of vascular endothelial miRs in circulating endothelial MPs with potential implications on vascular heath.

## Additional file

10.1186/s12933-016-0367-8 Sorting of microparticles (MPs) from plasma of diabetic patients by flow cytometry A MPs were gated according to their size and granularity. B Representative flow cytometry plot displaying sorting of MPs from the diabetic patients plasma using CD31-PE and CD42b-APC staining. **Figure S2.** Endothelial cell-derived (CD31+/CD42b-), platelet-derived (CD31+/CD42b+), and other cell-derived MPs (CD31-/CD42b-) were sorted from 10 plasma samples of DM patients, and miR-199a and miR-let7d expression were analyzed in MPs subspecies. Relative quantification of miR expression were determined using the comparative CT method [2-ddCT, internal control: Cel-miR-39]. MPs indicate microparticles.**Figure S3.** miR-21, miR-30, miR-92a, miR-139 and miR-222 were analyzed in ECs, hgECs, EMPs and hgEMPs. RNU6b served as endogenous control. *p<0.05, n=5-6.

## Abbreviations

miRs: microRNAs
MPs: microparticles
EMPs: endothelial microparticles
CV: cardiovascular
CAD: coronary artery disease
DM: diabetes mellitus
RNase: ribonuclease
DM: diabetes mellitus
NDM: non-diabetic mellitus
EDTA: ethylenediaminetetraacetic acid
HCAEC: human coronary artery endothelial cells
hgEMP: high glucose EMP
PBS: phosphate buffered saline
hgHCAEC: high glucose HCAEC
PCR: polymerase chain reaction
