# Supplementary figures and images for: Vascular endothelial microparticles-incorporated microRNAs are altered in patients with diabetes mellitus
Source: Cardiovasc Diabetol. 2016 Mar 22;15:49. doi: 10.1186/s12933-016-0367-8 (PMC4804519; doi:10.1186/s12933-016-0367-8)

## Slide 1
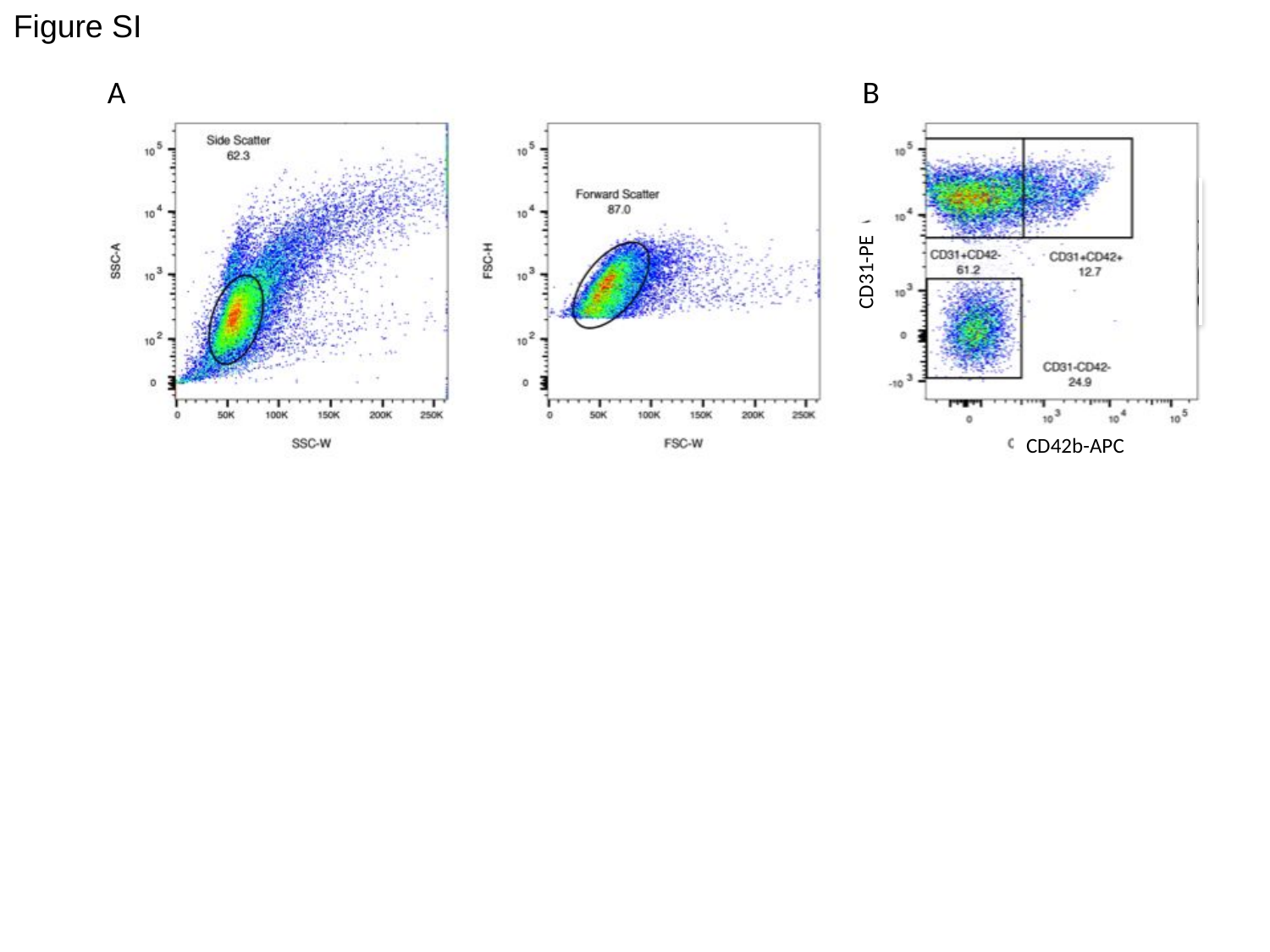

Figure SI
A
B
CD31-PE
CD42b-APC

## Slide 2
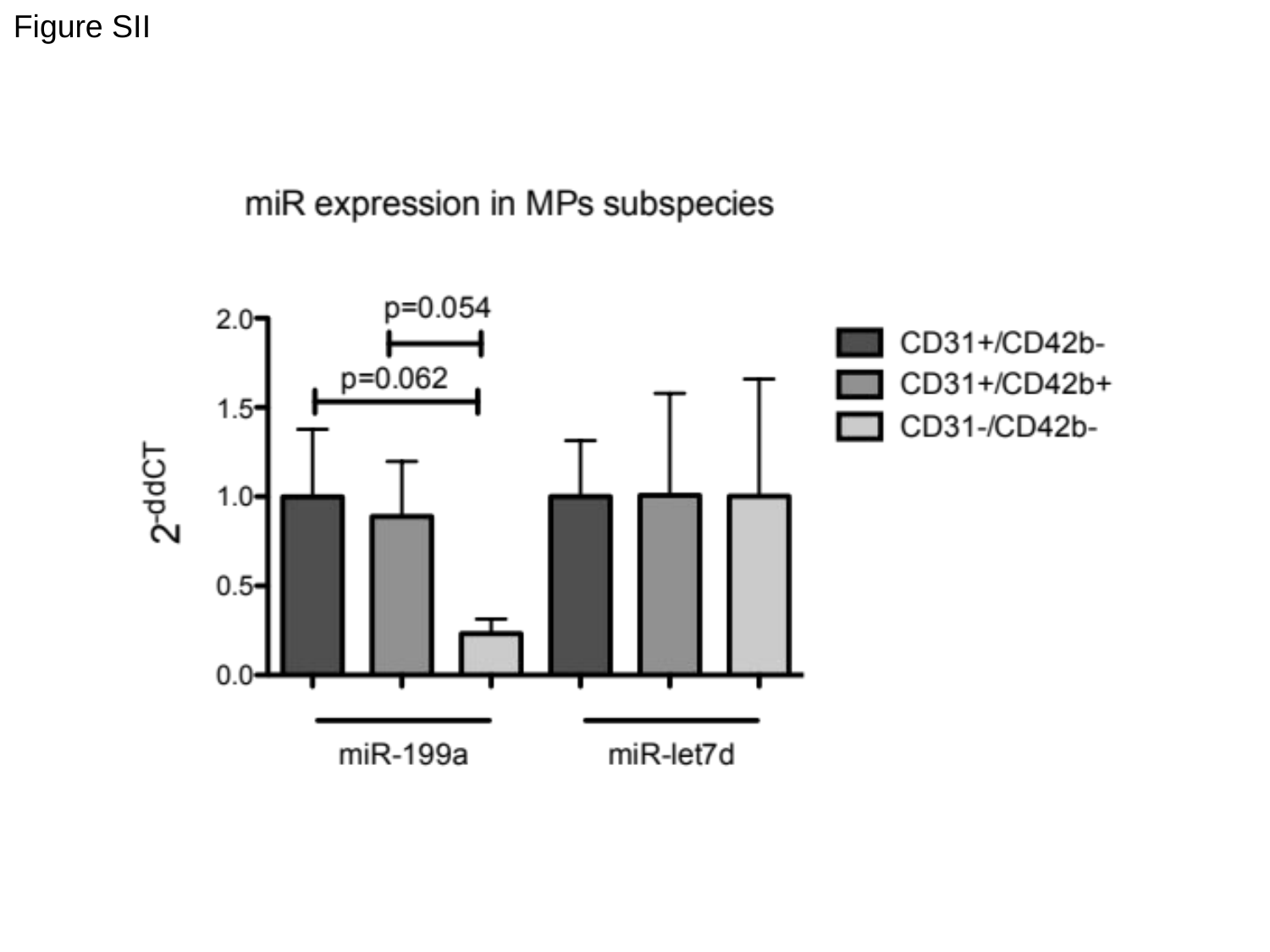

Figure SII

## Slide 3
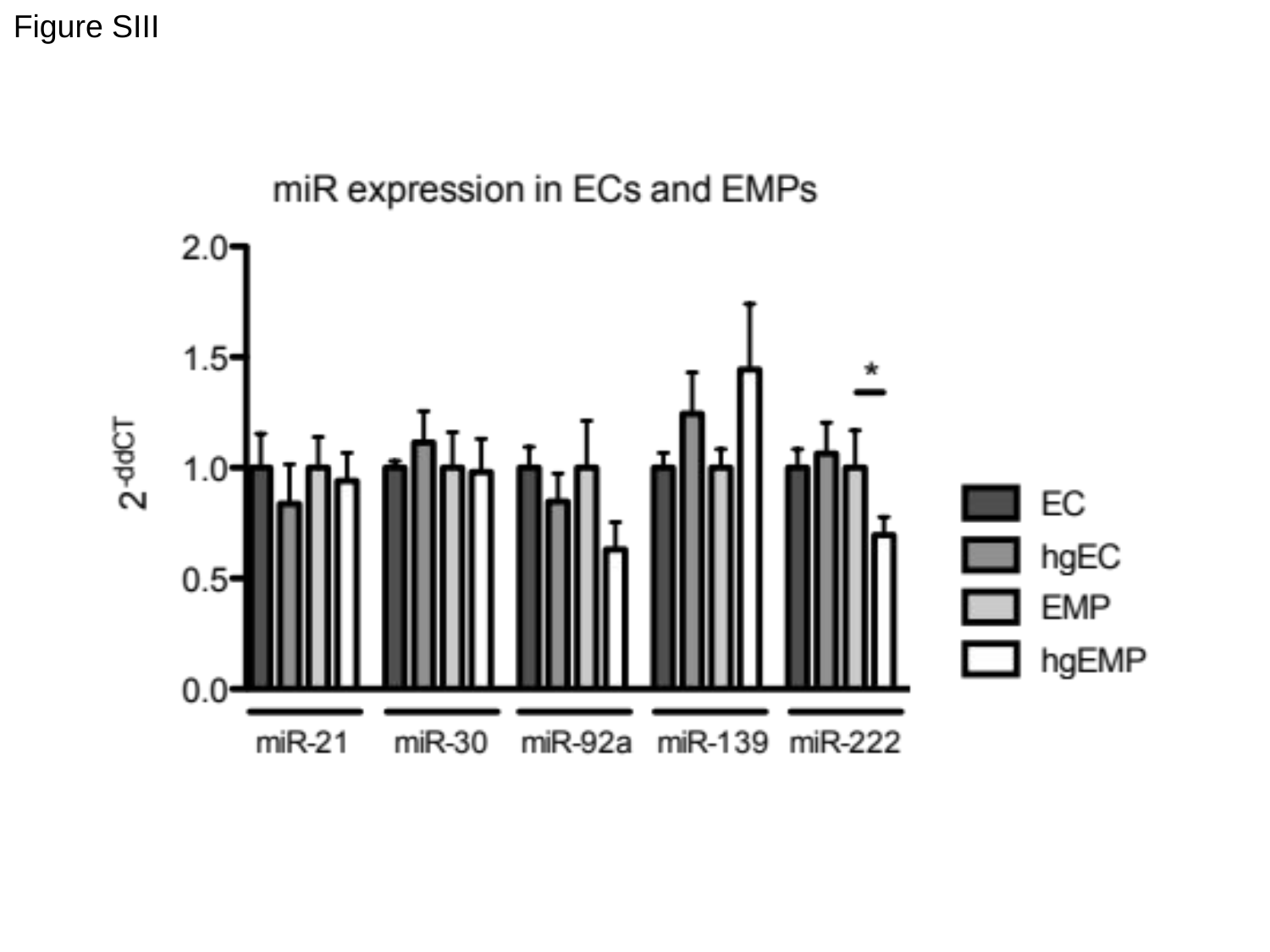

Figure SIII

Supplement: Supplementary file 1 — 10.1186/s12933-016-0367-8 Sorting of microparticles (MPs) from plasma of diabetic patients by flow cytometry A MPs were gated according to their size and granularity. B Representative flow cytometry plot displaying sorting of MPs from the diabetic patients plasma using CD31-PE and CD42b-APC staining. Figure S2. Endothelial cell-derived (CD31+/CD42b-), platelet-derived (CD31+/CD42b+), and other cell-derived MPs (CD31-/CD42b-) were sorted from 10 plasma samples of DM patients, and miR-199a and miR-let7d expression were analyzed in MPs subspecies. Relative quantification of miR expression were determined using the comparative CT method [2-ddCT, internal control: Cel-miR-39]. MPs indicate microparticles.Figure S3. miR-21, miR-30, miR-92a, miR-139 and miR-222 were analyzed in ECs, hgECs, EMPs and hgEMPs. RNU6b served as endogenous control. *p<0.05, n=5-6. [file 12933_2016_367_MOESM1_ESM.pptx]
